# Correction: Predicting the influence of climate on grassland area burned in Xilingol, China with dynamic simulations of autoregressive distributed lag models

**DOI:** 10.1371/journal.pone.0245828

**Published:** 2021-01-19

**Authors:** Ali Hassan Shabbir, Jiquan Zhang, James D. Johnston, Samuel Asumadu Sarkodie, James A. Lutz, Xingpeng Liu

## Notice of republication

This article was republished on December 30, 2020, to remove Fig 5 because this figure was derived from Fig 1 of [2], which was published in 2017 by the Midwest Political Science Association and is not offered under a CC-BY license. The *PLOS ONE* article [1] now cites [2] as a reference for the ARDL model used in the study, and Figs 6–8 have been renumbered as Figs 5–7.

In the revised article, citations have also been added to the Stationarity test subsection of the Methods and to the Discussion to address the following text overlap concerns.

- The ‘Stationary test’ subsection includes text that overlaps with [3, 4].
- In the Discussion, the following text overlaps with [5]: “Major fires are dependent on… during severe fire weather conditions.”

Please download this article again to view the correct version. The originally published version of this article, with Fig 5 redacted, and the republished, corrected article are provided here for reference.

In addition, it was noted that there is overlap in text, figures, and tables between this article [1] and an article published shortly thereafter in *Rangeland Ecology & Management* [6]. The two articles address the same overall research objective using similar methods and report overlapping results and conclusions. The authors commented that the *PLOS ONE* article included variables that were not included in [6] and used multiple models for the analyses whereas the second article reported results based on a single equation model. The *Rangeland Ecology & Management* article has been withdrawn at the authors’ request [6].

## Supporting information

S1 File Original article with Fig 5 redacted. (PDF)

S2 File Republished, corrected article. (PDF)
