# Supplementary material for: Correction: Predicting the influence of climate on grassland area burned in Xilingol, China with dynamic simulations of autoregressive distributed lag models
Source: PLoS One. 2021 Jan 19;16(1):e0245828. doi: 10.1371/journal.pone.0245828 (PMC7815123; doi:10.1371/journal.pone.0245828)
Supplement: S1 File — (PDF) [file pone.0245828.s001.pdf]

PLOS ONE

RESEARCH ARTICLE

Predicting the influence of climate on grassland area burned in Xilingol, China with dynamic simulations of autoregressive distributed lag models

Ali Hassan Shabbir[1,2,3☯], Jiquan Zhang[1,2,3]*, James D. Johnston[4☯], Samuel Asumadu Sarkodie[5‡], James A. Lutz[6‡], Xingpeng Liu[1,2,3‡]

1 Institute of Natural Disaster Research, School of Environment, Northeast Normal University, Changchun, China, **2** State Environmental Protection Key Laboratory of Wetland Ecology and Vegetation Restoration, Northeast Normal University, Changchun, China, **3** Laboratory for Vegetation Ecology, Ministry of Education, Changchun, China, **4** College of Forestry, Oregon State University, Oregon, United States of America, **5** Nord University Business School (HHN), Norway, **6** Wildland Resources Department, Utah State University, Utah, United States of America

☯ These authors contributed equally to this work.
‡ These authors also contributed equally to this work.
* zhangjq022@nenu.edu.cn

Check for updates

OPEN ACCESS

Citation: Shabbir AH, Zhang J, Johnston JD, Sarkodie SA, Lutz JA, Liu X (2020) Predicting the influence of climate on grassland area burned in Xilingol, China with dynamic simulations of autoregressive distributed lag models. PLoS ONE 15(4): e0229894. https://doi.org/10.1371/journal.pone.0229894

Editor: Delei Li, Institute of Oceanology Chinese Academy of Sciences, CHINA

Received: October 6, 2019

Accepted: February 18, 2020

Published: April 3, 2020

Copyright: © 2020 Shabbir et al. This is an open access article distributed under the terms of the Creative Commons Attribution License, which permits unrestricted use, distribution, and reproduction in any medium, provided the original author and source are credited.

Data Availability Statement: All relevant data are within the paper and its Supporting Information files.

Funding: This work was supported by the International (Regional) Cooperation and Exchange Programs of National Natural Science Foundation of China under grant no. 41961144019. The funder had no role in study design, data collection and analysis, decision to publish, or preparation of the manuscript.

Abstract

The influence of climate change on wildland fire has received considerable attention, but few studies have examined the potential effects of climate variability on grassland area burned within the extensive steppe land of Eurasia. We used a novel statistical approach borrowed from the social science literature—dynamic simulations of autoregressive distributed lag (ARDL) models—to explore the relationship between temperature, relative humidity, precipitation, wind speed, sunlight, and carbon emissions on grassland area burned in Xilingol, a large grassland-dominated landscape of Inner Mongolia in northern China. We used an ARDL model to describe the influence of these variables on observed area burned between 2001 and 2018 and used dynamic simulations of the model to project the influence of climate on area burned over the next twenty years. Our analysis demonstrates that area burned was most sensitive to wind speed and temperature. A 1% increase in wind speed was associated with a 20.8% and 22.8% increase in observed and predicted area burned respectively, while a 1% increase in maximum temperature was associated with an 8.7% and 9.7% increase in observed and predicted future area burned. Dynamic simulations of ARDL models provide insights into the variability of area burned across Inner Mongolia grasslands in the context of anthropogenic climate change.

Introduction

There is strong evidence that climate change and altered fuel characteristics associated with human activity can dramatically influence area burned by wildfire at large spatial scales [1, 2].

Competing interests: The authors have declared that no competing interests exist.

Understanding how climate influences fire in grasslands is challenging because of complex interactions between climate parameters, ignitions, and past fire history [3–13]. Previous research has demonstrated complex relationships between fire activity and climate variability in the extensive grasslands of Xilingol in northern China [14, 15]. Fire in this region potentially creates feedbacks between climate and fire occurrence both by climate forcing related to carbon emissions from fires and changes in flammability related to post-fire succession [16, 17]. The climate thresholds that potentially accelerate area burned in the Xilingol region remain poorly resolved. Identifying these thresholds is important to managers seeking to optimize the production of key ecosystem services from grasslands.

Many investigations of climate influence on fire extent rely on ordinary least squares (OLS) regression techniques [18, 19]. The OLS method is appropriate for analysis of stationary time-series—series in which the mean, variance, and autocorrelation structure are constant over time. In the case of non-stationary time series data, application of OLS may result in spurious relationships between variables [20, 21].

The goal of this study is to provide a comprehensive analysis of fire-climate relationships that accounts for potential non-stationarity in the time series analyzed and that distinguishes between "short-run" perturbations that move the time series analyzed apart over relatively short timeframes and "long-run" relationships in which relationships exhibit equilibrium over time. We accomplish this goal by implementing a method that is increasingly popular in social science investigations of political and economic trends—dynamic simulations of Autoregressive Distributed Lag (ARDL) models [22]. This method conveys results by constructing counterfactual scenarios to describe, in our case, the effects on grassland area burned by perturbations in climate and carbon emissions [23]. We provide a detailed description of the implementation of these methods. Although this analysis focused on the steppe lands of northern China, this methodology will be applicable to other investigations of broad-scale climate-fire relationships. Our results will provide a better understanding of the role of anthropogenic climate change on fire and help identify adaptation strategies.

Methods

Study area and data

Xilingol is located within the Autonomous Region of Inner Mongolia in northern China (Fig 1). The climate of this region is semi-arid, and maximum temperatures have increased by approximately 1.5˚C over the last 70 years. The extensive grasslands of this region burn mostly in the months of April, May, and September (Fig 2). Between 2001 and 2018 there were 832 grassland fires covering a total area of 42,190 ha (Fig 1). Carbon emissions from these fires and other sources in Xilingol may potentially influence area burned in this region both by contributing to atmospheric climate forcing of temperature, and because the fires that caused these emissions reset succession which potentially influences future flammability (Fig 3).

We investigated the relationship between area burned and seven variables: monthly averages of minimum and maximum temperature (in degrees C), monthly average relative humidity (percentage), monthly average precipitation (in millimeters), average monthly wind speed (in meters per second), average monthly carbon emissions from fires in Xilingol (in grams centimeter^2), and average sunlight (in hours per month) (Fig 4). All climate data were obtained from the China Meteorological Data Sharing Service Center (http://www.cdc.cma.gov.cn/) for the period 2001 to 2018. Data for the number of hectares of grassland area burned and carbon emissions from fire and other sources were acquired from the Monitoring Center of the Ministry of Agriculture. (http://www.moa.gov.cn/). We combined biomass consumed in fire and carbon emissions in the models we describe below. We log-transformed the data to address

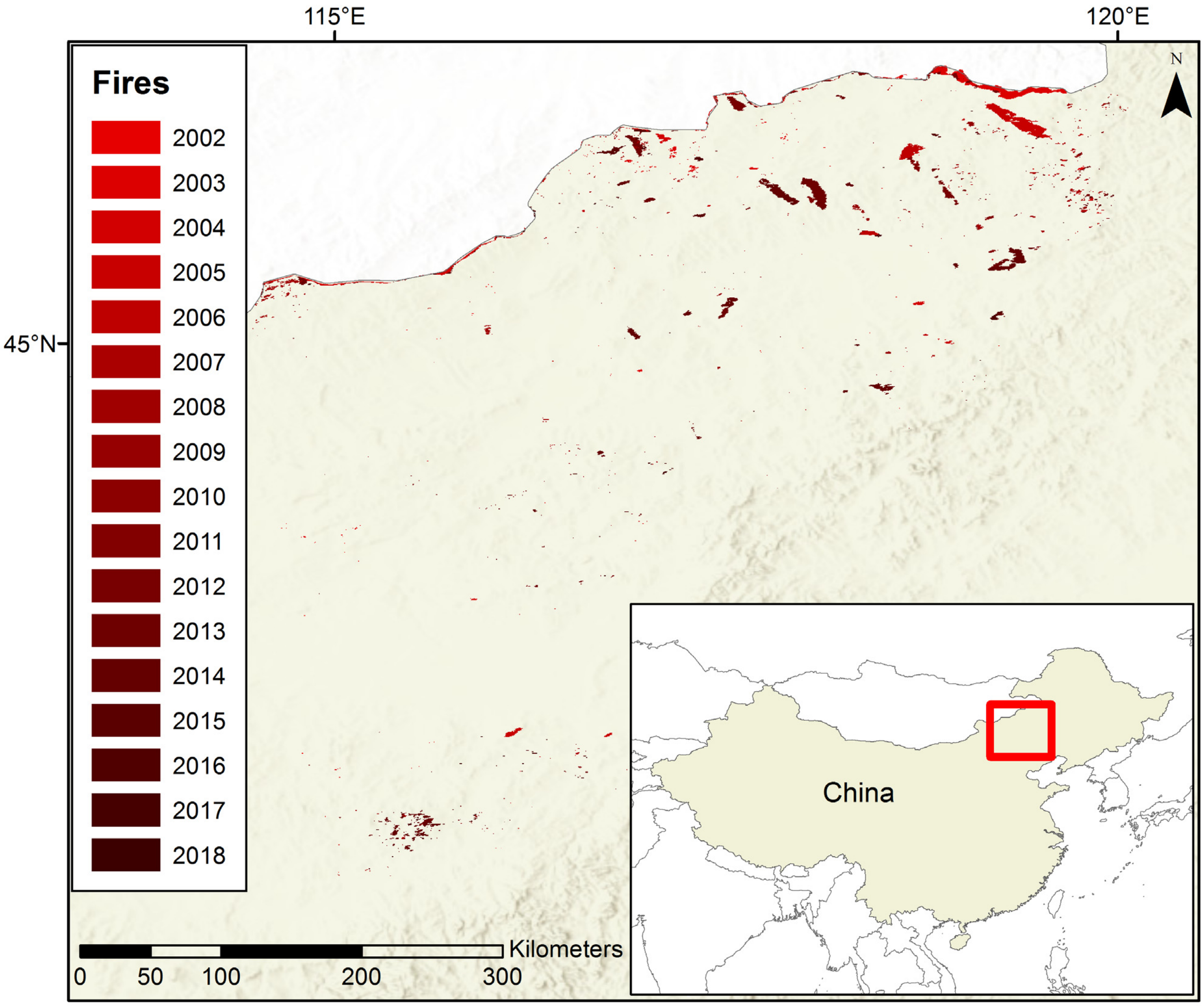

Fig 1. The study area Xilingol outlined in red with the area of grassland fires in each year from 2002–2018.

https://doi.org/10.1371/journal.pone.0229894.g001

heteroscedasticity [24], multi-collinearity [25] and assessed autocorrelation using the Durbin-Watson statistic [26, 27]. Dynamic simulations of ARDL models were performed using the Stata module [28].

Stationarity test

We graphically illustrate the workflow of implementing ARDL models in Fig 5. The first step in the analysis of potentially non-stationary time series data is an Augmented Dickey-Fuller (ADF) test to investigate the order of integration of variables [29]. If a series has constant mean and variance it is represented as I(0) and we call it a stationary series. A nonstationary series has a changing mean and variance which can be made stationary by taking the first difference or second difference of the series denoted as I(1) and I(2). We used the following functional form for the ADF test from Gujarati and Porter [30]:

$$\Delta X_t = \alpha_0 + \alpha_1 t + \lambda X_{t-1} + \sum_{t=1}^{m} \phi_t \Delta X_{t-1} + u_t \quad (1)$$

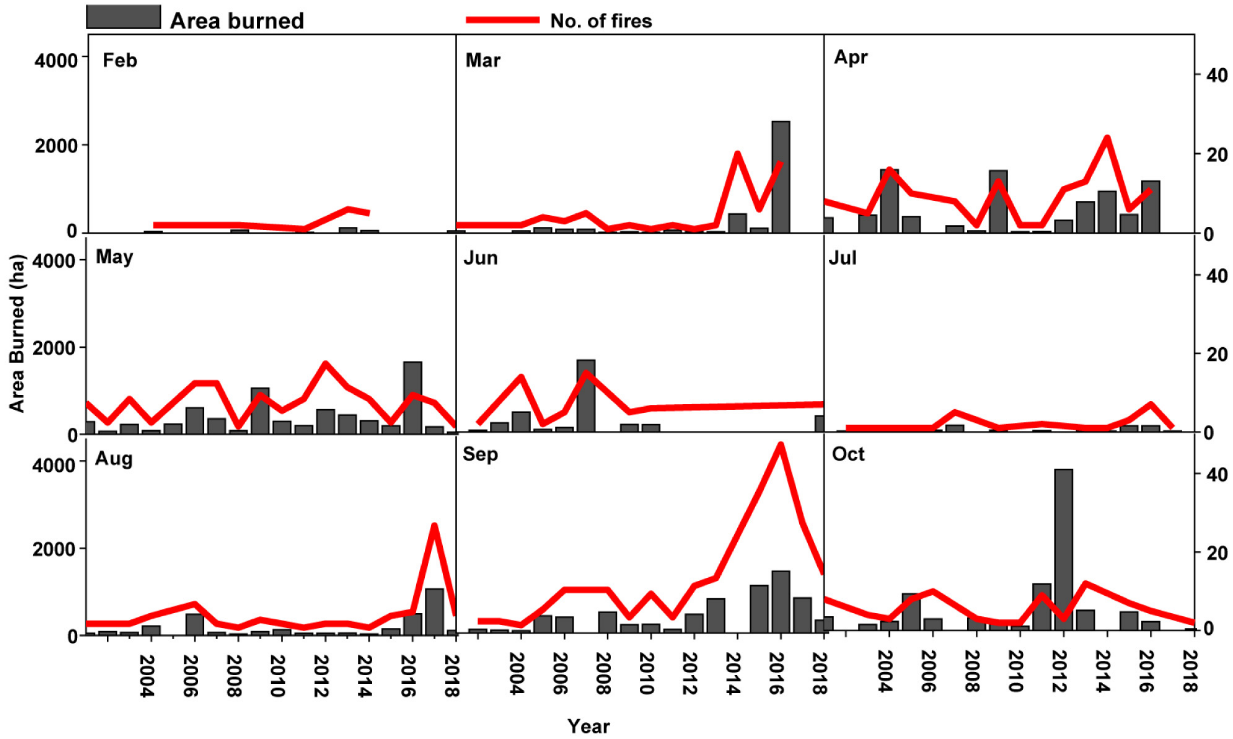

Fig 2. The total area burned (left y-axis) and the number of fires (right y-axis) by month between 2001 and 2018 in Xilingol League, China. Grey bars indicate area burned and red lines indicate the number of fires. There were no fires in January, November, or December between 2001 and 2018.

https://doi.org/10.1371/journal.pone.0229894.g002

Where X_t represents the variable series, Δ represents the first difference, and the lagged difference terms are included to correct for serial correlations of the disturbance terms on the right side of the equation. The Schwarz information criterion (SIC) was used for the selection of lagged differences. When $\theta = 0$, the X_t variable series has a unit root and an I(1) process governed by a stochastic trend. If the selected time series variable appears to be integrated of order one, the investigation of 2nd order unit root is performed by using the following expression:

$$\Delta^2 X_t = \beta_0 + \theta \Delta X_{t-1} + \sum\nolimits_{t=1}^{w} \gamma_t \Delta^2 X_{t-1} + \varepsilon_t \quad (2)$$

where Δ^2 represents the second-difference operator. The variable X_t is integrated of order two or I(2) if $\gamma = 0$. Suppose d shows the number of times that the variable Xt must be differenced to become stationary, then the series X_t is integrated of order d or I(d). If the ADF test statistic value was higher than the critical values at a 5 percent significance level, we considered it to be a stationary series but if the test statistic value is lower than the critical values, then, we classified it as a nonstationary series. If non-stationary, the series was differenced to make it stationary. Providing variables are either integrated of order *I(0)* or integrated of order *I(1)* or both but not *I(2)*, it is possible to estimate an ARDL model in error-correction form and perform a bounds test for cointegration of variables.

Bounds test

The dynamic simulated ARDL approach employs a bounds test to check the long-run relationship between variables. During bounds testing, a long-run relationship between variables exists

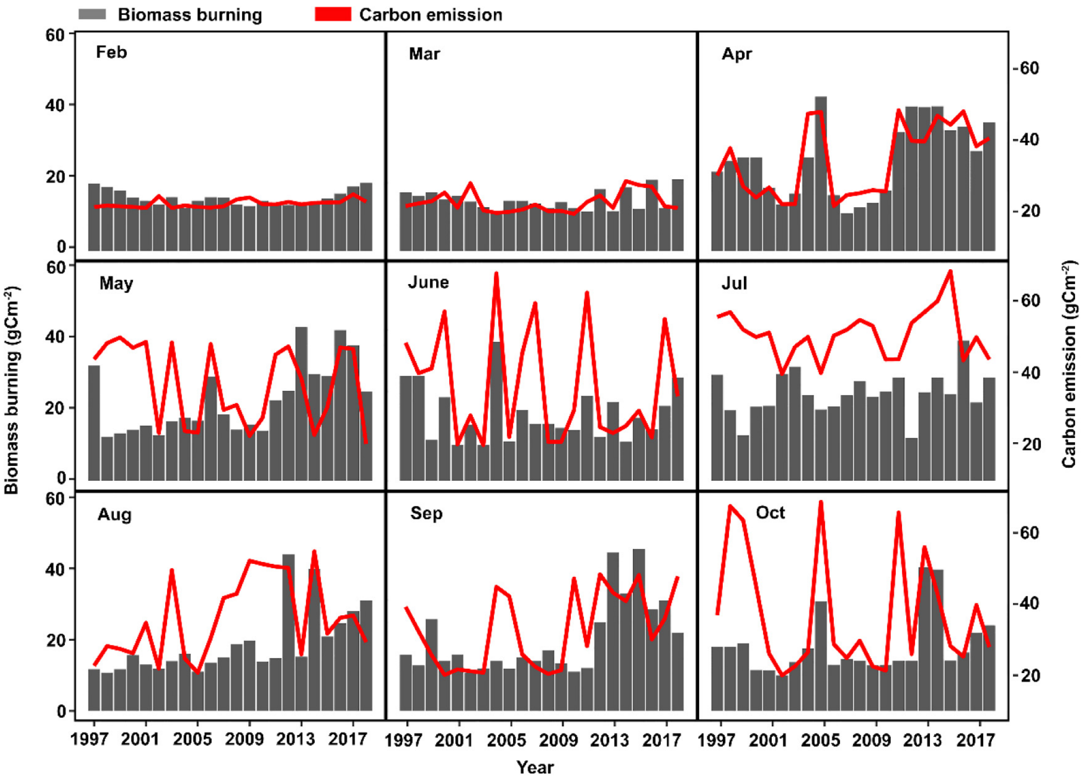

Fig 3. Carbon emissions (left y-axis) from all sources and biomass burning (right y-axis) by month between 2001 and 2018 in Xilingol League, China. Grey bars indicate biomass burning and red lines indicate carbon emissions. There were no fires in January, November, or December between 2001 and 2018.

https://doi.org/10.1371/journal.pone.0229894.g003

if the F-statistic is greater than the upper bound critical value at a 5 percent significance level. If the F-statistic is less than the upper bound critical value, then the null hypothesis of no long-run relationship between variables cannot be rejected. If the F-statistic value lies between the upper and lower bound critical values, a decision about co-integration remains inconclusive. The null hypothesis of no long-run relationship is represented as H_0: δ1 = δ2 = δ3 = δ4 = δ5 = δ6 = δ7 = δ8 = 0 tested during the model estimation. The ARDL bounds testing estimation model follows:

$$\begin{aligned}\Delta\, ln\ area\ burned &= \alpha + \delta_1\, ln\ area\ burned_{t-i} + \delta_2\, ln\ Rel.humidity_{t-i} + \\ &\delta_3\, ln\ Sunlight_{t-i} + \delta_4\, ln\ Tmax_{t-i} + \delta_5\, ln\ Tmin_{t-i} + \delta_6\, ln\ Cem_{t-i} + \delta_7\, ln\ Wind_{t-i} + \\ &\delta_8\, ln\ Rainfall_{t-i} + \sum\nolimits_{i=1}^{n} \beta_2 \Delta\, ln\ \ area\ burned_{t=i} + \sum\nolimits_{i=1}^{n} \beta_3 \Delta\, ln\ \ humidity_{t=i} + \\ &\sum\nolimits_{i=1}^{n} \beta_4 \Delta\, ln\ \ Sunlight_{t=i} + \sum\nolimits_{i=1}^{n} \beta_5 \Delta\, ln\ \ Tmax_{t=i} + \sum\nolimits_{i=1}^{n} \beta_6 \Delta\, ln\ \ Tmin_{t=i} + \\ &\sum\nolimits_{i=1}^{n} \beta_7 \Delta\, ln\ Cem_{t=i} + \sum\nolimits_{i=1}^{n} \beta_8 \Delta\, ln\ \ Wind_{t=i} + \sum\nolimits_{i=1}^{n} \beta_9 \Delta\, ln\ \ Rainfall_{t=i} + e_t\end{aligned} \tag{3}$$

Where Δ is the change operator, ln is its natural logarithm, and t-i is the optimal number of lags selection based on Schwartz Bayesian information criterion and Akaike information criterion. Delta (δ) and beta (β) are the parameters to be estimated. If a long-run relationship is

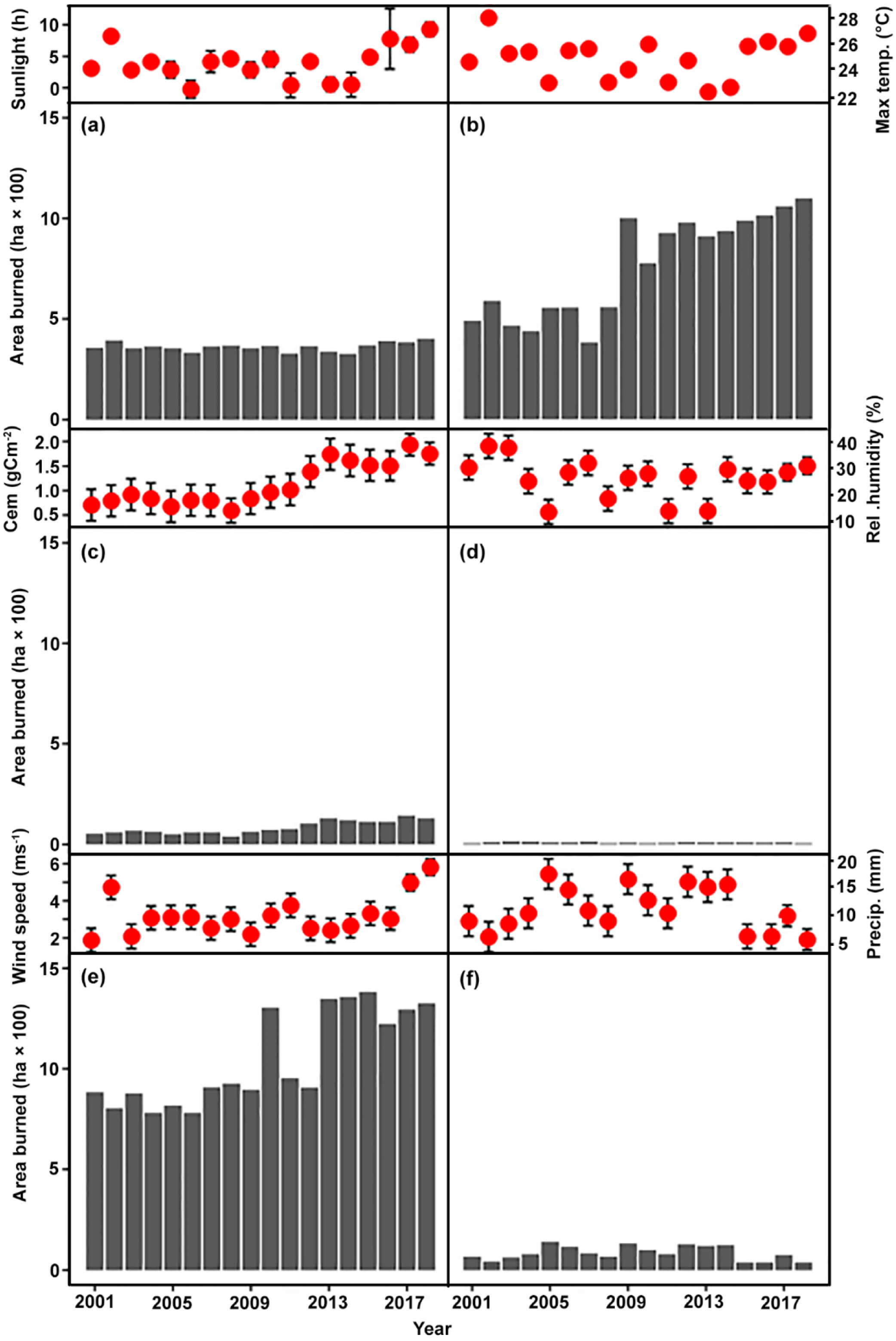

Fig 4. A relationship between area burned and climate variables between 2001 and 2018 in Xilingol League, China. The area burned is shown with grey bars on the bottom panels and climate variables are shown with red points on the upper panels (see text).

https://doi.org/10.1371/journal.pone.0229894.g004

Fig 5. Flowchart for an implementation of the Autoregressive Distributed Lag (ARDL) model.

https://doi.org/10.1371/journal.pone.0229894.g005

found, short- and long-run elasticities can be estimated using dynamic simulations of an ARDL model.

After confirming the long-run relationship among the variables, we incorporated the cumulative sum (CUSUM) and cumulative sum of squares (CUSUMSQ) tests developed by Brown et al. [31] to check the goodness of fit for the ARDL model [32]. These tests are performed on the residuals of the error correction model and reported in graphical form. Stable models fall within the 5% critical bound of CUSUM and CUSUMQ plots.

Dynamic simulated ARDL models. We selected the dynamic simulation ARDL model with cointegrated variables embedded in a vector autoregressive time series model (VAR) [33]. This method is designed to estimate the effect of a group of explanatory variables on a single response (area burned) with variable measurements taken discretely over time (also known as a single equation model framework). In contrast to ordinary least squares (OLS) regression, both current and lagged values of explanatory variables are considered in an ARDL framework and the estimated effect on the response can be instantaneous or observed gradually on future time steps. The error correction algorithm of the dynamic ARDL technique was used to

develop eight models via 5000 simulations of the vector of parameters from a multivariate normal distribution [34]. We selected the best regression model using dynamic simulations ARDL model with some methodological modifications introduced with the general formulation expressed as follows:

$$\Delta(y)_t = \alpha_0 + \theta_0(y)_{t-1} + \theta_1(x_1)_{t-1} + \cdots + \theta_k(x_k)_{t-1} + \sum_{i=1}^{p} (\alpha_i)\Delta(y)_{t-1} + \sum_{j=0}^{q1} \beta_{1-j}\Delta (x_1)_{t-j} + \cdots + \sum_{j=0}^{qk} \beta_{k-j}\Delta (x_k)_{t-j} + \varepsilon_t, \tag{4}$$

Where $\Delta\, y$ represents a change in the exogenous variable, α_0 is the intercept of all exogenous variables at time $t - 1$, which affects the level of maximum lagged p and qk to first-differences Δ with error term ε on the response of t. The null hypothesis of a level relationship is assessed using Kripfganz and Schneider [35] critical values and approximate p-values based on response surface regressions. To reject the null hypothesis of no level relationship $H_0 = \theta_0 + \theta_1 + \cdots + \theta_k = 0$, the F-statistic from the jointly zero estimation of all parameters on the climate threshold variables in level and the lagged grassland area burned coefficient must be above the upper bound [I(1)] critical values. The empirical specification in Eq (4) can be re-written into eight conceptual models as:

MODEL 1:

$$\begin{aligned}&\Delta\ ln(area\ burned)_t = \alpha_0 + \theta_0\ ln(area\ burned)_{t-1} +\\ &\beta_1\Delta \ln(Cem/biobur)_t + \theta_1 \ln(Cem/biobur)_{t-1} + \beta_2\Delta \ln(Rel.humidity)_t +\\ &\theta_2 \ln(Rel.humidity)_{t-1} + \beta_3\Delta \ln(Tmin)_t + \theta_3 \ln(Tmin)_{t-1} + \beta_4\Delta \ln(Tmax)_t +\\ &\theta_4 \ln(Tmax)_{t-1} + \beta_5\Delta \ln(Precip)_t + \theta_5 \ln(Precip)_{t-1} + \beta_6\Delta \ln(Sunlight)_t +\\ &\theta_6 \ln(Sunlight)_{t-1} + \beta_7\Delta \ln(Wind)_t + \theta_7 \ln(Wind)_{t-1} + u_t,\end{aligned} \tag{5}$$

MODEL 2:

$$\begin{aligned}&\Delta\ ln(Cem/biobur)_t = \alpha_0 + \theta_0\ ln(Cem/biobur)_{t-1} + \beta_1\Delta \ln(area\ burned)_t +\\ &\theta_1 \ln(area\ burned)_{t-1} + \beta_2\Delta \ln(Rel.humidity)_t + \theta_2 \ln(Rel.humidity)_{t-1} +\\ &\beta_3\Delta \ln(Tmin)_t + \theta_3 \ln(Tmin)_{t-1} + \beta_4\Delta \ln(Tmax)_t + \theta_4 \ln(Tmax)_{t-1} + \beta_5\Delta \ln(Precip)_t +\\ &\theta_5 \ln(Precip)_{t-1} + \beta_6\Delta \ln(Sunlight)_t + \theta_6 \ln(Sunlight)_{t-1} + \beta_7\Delta \ln(Wind)_t +\\ &\theta_7 \ln(Wind)_{t-1} + u_t,\end{aligned} \tag{6}$$

MODEL 3:

$$\begin{aligned}&\Delta\ ln(Rel.humidity)_t = \alpha_0 + \theta_0\ ln(Rel.humidity)_{t-1} +\\ &\beta_1\Delta \ln(Cem)_t + \theta_1 \ln(Cem/biobur)_{t-1} + \beta_2\Delta \ln(area\ burned)_t + \theta_2 \ln(area\ burned)_{t-1} +\\ &\beta_3\Delta \ln(Tmin)_t + \theta_3 \ln(Tmin)_{t-1} + \beta_4\Delta \ln(Tmax)_t + \theta_4 \ln(Tmax)_{t-1} + \beta_5\Delta \ln(Precip)_t +\\ &\theta_5 \ln(Precip)_{t-1} + \beta_6\Delta \ln(Sunlight)_t + \theta_6 \ln(Sunlight)_{t-1} + \beta_7\Delta \ln(Wind)_t +\\ &\theta_7 \ln(Wind)_{t-1} + u_t,\end{aligned} \tag{7}$$

MODEL 4:

$$\begin{aligned}&\Delta\ ln(Tmin)_t = \alpha_0 + \theta_0\ ln(Tmin)_{t-1} + \beta_1\Delta\ln(Cem)_t + \\&\theta_1\ln(Cem/biobur)_{t-1} + \beta_2\Delta\ln(Rel.humidity)_t + \theta_2\ln(Rel.humidity)_{t-1} + \\&\beta_3\Delta\ln(area\ burned)_t + \theta_3\ln(area\ burned)_{t-1} + \beta_4\Delta\ln(Tmax)_t + \theta_4\ln(Tmax)_{t-1} + \\&\beta_5\Delta\ln(Precip)_t + \theta_5\ln(Precip)_{t-1} + \beta_6\Delta\ln(Sunlight)_t + \theta_6\ln(Sunlight)_{t-1} + \\&\beta_7\Delta\ln(Wind)_t + \theta_7\ln(Wind)_{t-1} + u_t,\end{aligned} \tag{8}$$

MODEL 5:

$$\begin{aligned}&\Delta\ ln(Tmax)_t = \\&\alpha_0 + \theta_0\ ln(Tmax)_{t-1} + \beta_1\Delta\ln(Cem)_t + \theta_1\ln(Cem/biobur)_{t-1} + \beta_2\Delta\ln(Rel.humidity)_t + \\&\theta_2\ln(Rel.humidity)_{t-1} + \beta_3\Delta\ln(Tmin)_t + \theta_3\ln(Tmin)_{t-1} + \beta_4\Delta\ln(area\ burned)_t + \\&\theta_4\ln(area\ burned)_{t-1} + \beta_5\Delta\ln(Precip)_t + \theta_5\ln(Precip)_{t-1} + \beta_6\Delta\ln(Sunlight)_t + \\&\theta_6\ln(Sunlight)_{t-1} + \beta_7\Delta\ln(Wind)_t + \theta_7\ln(Wind)_{t-1} + u_t,\end{aligned} \tag{9}$$

MODEL 6:

$$\begin{aligned}&\Delta\ ln(Precip)_t = \\&\alpha_0 + \theta_0\ ln(Precip)_{t-1} + \beta_1\Delta\ln(Cem)_t + \theta_1\ln(Cem/biobur)_{t-1} + \beta_2\Delta\ln(Rel.humidity)_t + \\&\theta_2\ln(Rel.humidity)_{t-1} + \beta_3\Delta\ln(Tmin)_t + \theta_3\ln(Tmin)_{t-1} + \beta_4\Delta\ln(Tmax)_t + \theta_4\ln(Tmax)_{t-1} + \\&\beta_5\Delta\ln(area\ burned)_t + \theta_5\ln(area\ burned)_{t-1} + \beta_6\Delta\ln(Sunlight)_t + \theta_6\ln(Sunlight)_{t-1} + \\&\beta_7\Delta\ln(Wind)_t + \theta_7\ln(Wind)_{t-1} + u_t,\end{aligned} \tag{10}$$

MODEL 7:

$$\begin{aligned}&\Delta\ ln(Sunlight)_t = \alpha_0 + \theta_0\ ln(Sunlight)_{t-1} + \beta_1\Delta\ln(Cem)_t + \theta_1\ln(Cem/biobur)_{t-1} + \beta_2 \\&\beta_2\Delta\ln(Rel.humidity)_t + \theta_2\ln(Rel.humidity)_{t-1} + \beta_3\Delta\ln(Tmin)_t + \theta_3\ln(Tmin)_{t-1} + \\&\beta_4\Delta\ln(Tmax)_t + \theta_4\ln(Tmax)_{t-1} + \beta_5\Delta\ln(Precip)_t + \theta_5\ln(Precip)_{t-1} + \beta_6\Delta\ln(area\ burned)_t + \\&\theta_6\ln(area\ burned)_{t-1} + \beta_7\Delta\ln(Wind)_t + \theta_7\ln(Wind)_{t-1} + u_t,\end{aligned} \tag{11}$$

MODEL 8:

$$\begin{aligned}&\Delta\ ln(Wind)_t = \\&\alpha_0 + \theta_0\ ln(Wind)_{t-1} + \beta_1\Delta\ln(Cem)_t + \theta_1\ln(Cem/biobur)_{t-1} + \beta_2\Delta\ln(Rel.humidity)_t + \\&\theta_2\ln(Rel.humidity)_{t-1} + \beta_3\Delta\ln(Tmin)_t + \theta_3\ln(Tmin)_{t-1} + \beta_4\Delta\ln(Tmax)_t + \theta_4\ln(Tmax)_{t-1} + \\&\beta_5\Delta\ln(Precip)_t + \theta_5\ln(Precip)_{t-1} + \beta_6\Delta\ln(area\ burned)_t + \theta_6\ln(area\ burned)_{t-1} + \\&\beta_7\Delta\ln(Sunlight)_t + \theta_7\ln(Sunlight)_{t-1} + u_t,\end{aligned} \tag{12}$$

Where α_0 is the intercept, θ's and β's are the parameters to be estimated and u denotes the white noise at time t. All variables are taken in logarithmic scale to stabilize the variance and so that results can be presented as "elasticities" in which coefficients represent the estimated percent change in the burned area dependent variable for a percent change in a climate independent variable.

Results

ADF and PP unit root tests indicated that maximum temperature and carbon emissions were stationary at the 10% significance level presented (Table 1). All other variables were integrated at order one I(1). The lag selection criteria presented in Table 2 was used to select the optimal lag order for the ARDL model estimation technique. The lag selection criteria (final prediction error, Schwarz Bayesian Criterion, and Hannan-Quinn Information Criterion) confirmed lag one as the optimal lag for subsequent analysis. Table 3 shows the results of the ARDL bounds cointegration test. The F-statistic of the estimated models was above the upper bounds critical values, thus rejecting the null hypothesis of no level relationship. The absence of I(2) variable validated the application of ARDL bound testing technique.

Long-run coefficients of observed and predicted area burned

An ARDL model with lag (1,1,1,1,1,0,1) was selected based on the Schwarz Bayesian Criterion. The dependent variable (grassland area burned) and the regressors with 832 observations from 2001 to 2018 were estimated using dynamic simulations of an ARDL model. Wind speed, maximum temperature, and carbon emissions showed a significant relationship to observed and predicted area burned. All other variables tested were non-significant. A one percent increase in wind speed was associated with a 20.8% and 22.8% increase in observed and predicted area

Table 1. Unit root results.

		ADF test statistics		PP test statistics		Level of Significance
**Observed area burned Variables**	**Levels**	**1st Differences**	**Levels**	**1st Differences**	**1% Level of Significance**	**5% Level of Significance**
ln Cem	-6.35*	-7.941 **	-6.81 *	-14.001 **	1(1)	1(0)
ln Rel.humidity	-7.138	-6.922 **	-4.658	-24.824 **	1(1)	1(0)
ln T_{min}	3.880	-8.972 **	-4.864	-11.821 **	1(1)	1(1)
ln T_{max}	-3.44*	-13.52 **	-6.053 *	-24.936 **	1(1)	1(1)
ln Precip.	-7.668	-12.74 **	-9.874	-32.864 **	1(1)	1(1)
ln Sunlight	-9.612	-8.097 **	-7.826	-38.326 **	1(1)	1(1)
ln Wind Speed	-5.331	-7.166 **	-8.667	-27.942 **	1(1)	1(0)
**Predicted area burned**						
ln Cem	-9.35*	-6.248 **	-7.01 *	-13.981 **	1(1)	1(1)
ln Rel.humidity	-7.234	-7.121 **	-9.154	-21.029 **	1(1)	1(1)
ln T_{min}	6.582	-8.176 **	-4.861	-16.127 **	1(1)	1(0)
ln T_{max}	-6.04*	-17.71 **	-7.050 *	-21.034 **	1(1)	1(1)
ln Precip.	-5.160	-12.81 **	-7.074	-30.094 **	1(1)	1(0)
ln Sunlight	-6.010	-7.090 **	-9.121	-36.020 **	1(1)	1(0)
ln Wind Speed	-9.139	-8.660 **	-9.460	-20.049 **	1(1)	1(1)

Note:

* represents 10% significance level while

** represents 5% significance level

https://doi.org/10.1371/journal.pone.0229894.t001

Table 2. The lag selection criteria.

Lag	LogL	LR	FPE	AIC	SBC	HQ
0	131.06	NA	8.11e+11	-8.71	-9.11	-6.91
1	119.02	276.21	1.21e+11*	-14.34	-15.04*	-22.21*
2	129.10	63.75	6.02e+11	-13.01	-16.91	-23.67
3	114.14	91.02*	9.01e+11	-16.42*	-18.61	-24.64

Notes:

* indicates lag order selected by the criterion,

LogL: log-likelihood, LR: sequential modified log-ratio test statistic (each test at 5% level), FPE: Final prediction error, AIC: Akaike information criterion, SBC: Schwarz Bayesian Criterion, and HQ: Hannan-Quinn information criterion.

https://doi.org/10.1371/journal.pone.0229894.t002

burned respectively while holding other climatic variables constant. A one percent increase in maximum temperature was associated with 8.7% and 9.8% increase in observed and predicted area burned while holding other climatic variables constant. A one percent increase in carbon emission was associated with only a 2.6% and 2.8% increase in observed and predicted area burned with other variables held constant. The estimated long-run coefficients for the effect of climate variables on the grassland area burned are shown in Table 4 while a plot of the modelled values showing the influence of selected climate variables on area burned in Xilingol is shown in Fig 6.

Fig 7 shows the results of the dynamic stimulated ARDL model in which the grassland area response is predicted at various time steps after forcing a one standard deviation increment of each climate variable. These simulations showed that a one standard deviation increase in maximum temperature and wind speed would significantly increase area burned over a twenty-year period beginning in approximately ten years. Carbon emissions were associated with an increased area burned at the 75% confidence interval after ten years, but not at a 90% or 95% confidence interval.

Short-run coefficients of observed and predicted area burned

The short-run equilibrium relationship of observed and future area burned was examined using the error correction representation of the dynamic stimulated ARDL model with 832 observations over the period 2001 to 2018. The estimated elasticity of wind speed in the short run was found to be low relative to the long-run elasticity. A 1% increase in wind speed was associated with a 6.1% and 7.8% increase in observed and predicted area burned respectively

Table 3. ARDL bounds cointegration test.

Models	statistic		1(0)	1(1)	p-value 1(0)	1(1)
Lnareaburned = f (lnCem lnRel.humidity lnTmin lnTmax lnPrecip lnSunlight lnWind)	F	10.12	2.34	3.63	0.001	0.001
lnCem = f (lnareaburned lnRel.humidity lnTmin lnTmax lnPrecip lnSunlight lnWind)	F	9.71	2.62	3.84	0.001	0.001
lnRel.humidity = f (lnCem lnarea burned lnTmin lnTmax lnPrecip lnSunlight lnWind)	F	8.55	2.28	3.41	0.002	0.001
lnTmin = f (lnCem lnRel.humidity lnarea burned lnTmax lnPrecip lnSunlight lnWind)	F	11.22	2.91	4.10	0.001	0.006
lnTmax = f (lnCem lnRel.humidity lnTmin lnarea burned lnPrecip lnSunlight lnWind)	F	6.11	2.86	3.90	0.001	0.001
lnPrecip = f (lnCem lnRel.humidity lnTmin lnTmax lnarea burned lnSunlight lnWind)	F	10.63	2.74	3.25	0.008	0.007
lnSunlight = f (lnCem lnRel.humidity lnTmin lnTmax lnPrecip lnarea burned lnWind)	F	5.21	2.31	4.18	0.001	0.001
lnWind = f (lnCem lnRel.humidity lnTmin lnTmax lnPrecip lnarea burned lnSunlight)	F	8.44	2.11	3.71	0.001	0.001

https://doi.org/10.1371/journal.pone.0229894.t003

Table 4. Estimated long-run coefficients for the effect of climate variables on grassland area burned from 2001 to 2018 using dynamic simulations of ARDL models.

	Observed		Predicted	
**Regressor**	**Coefficient**	**Prob.**	**Coefficient**	**Prob.**
L_n Wind speed	20.81 *	0.001	22.76*	0.001
L_n T_{max}	8.651 *	0.001	9.746*	0.001
L_n T_{min}	1.026	0.401	1.064	0.517
L_n Rel.humidity	1.102	0.601	1.141	0.612
L_n Precip	1.421	0.032	1.914	0.063
L_n Sunlight	4.481	0.001	4.606	0.001
L_n Cem	2.616 **	0.001	2.814**	0.001
R^2	0.631		R^2	0.671
Adj. R^2	0.644		Adj. R^2	0.682
Number of simulations.	5000		Number of simulations.	5000
Sum squared Res.	0.061		Sum squared Res.	0.056
Durbin-Watson-stat	2.231		Durbin-Watson-stat	2.106
Diagnostics				
$\chi\ LM - ARCH^2$	0.41		$\chi\ LM - ARCH^2$	0.44
$\chi\ LM - B - G^2$	0.53		$\chi\ LM - B - G^2$	0.51
Functional Form Ramsey RESET test	0.41		Functional Form Ramsey RESET test	0.49
Normality	0.64		Normality	0.68

Note:

* denotes 1% significance level while

** denotes 5% significance level

https://doi.org/10.1371/journal.pone.0229894.t004

while holding other climatic variables constant. The estimated short-run elasticity of carbon emission was associated with a 1.8% and 1.9% increase in observed and predicted area burned while other climatic variables were held constant. The empirical results of the short-run equilibrium relationship are presented in Table 5 while the monthly correlation between grassland area burned and climatic variables are depicted in Table 6. The area burned exhibited stronger correlations with maximum temperature than carbon emissions. The correlation of wind speed and sunlight to area burned varied between months.

Model validation

Diagnostic tests are critical to examine the independence of the residuals of the estimated models. Several diagnostic tests such as the LM test for autoregressive conditional heteroskedasticity, Breusch-Godfrey test for autocorrelation, Ramsey RESET test for functional form and Jarque-Bera test for normality were employed to verify the estimated long- and short- run elasticities of the dynamic stimulated ARDL Model. Table 5 shows that the estimated models are free from heteroskedasticity, autocorrelation, functional misspecification and are normally distributed. Fig 8 presents the plots of the cumulative sum of recursive residuals for the dynamic stimulated ARDL model and indicates that values are within the 95% confidence bands—confirming the stability of the estimated models.

Discussion

This study demonstrates that in the long-run, grassland area burned in Xilingol is more sensitive to changes in wind speed and temperature than other climate variables [10, 15, 36]. Past

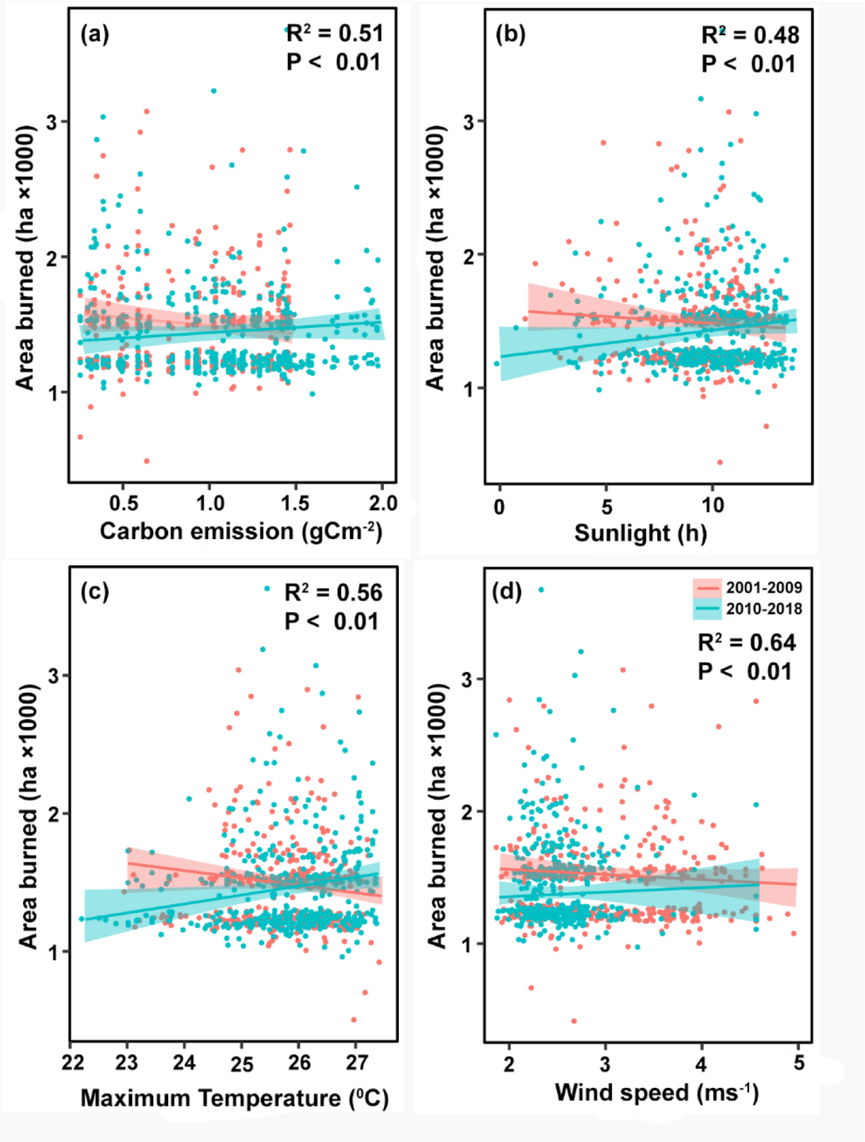

Fig 6. A plot of the modelled values showing the influence of variation in climatic variables on the area burned in Xilingol.

https://doi.org/10.1371/journal.pone.0229894.g006

fire history and the carbon emissions that resulted from these fires had a marginal influence on the projected future fire. Area burned is likely to increase given warmer winter and spring temperatures related to directional climate change [6].

Variability in precipitation, in contrast, had no significant effect on area burned, which is surprising because fuel moisture often plays a critical role in fire spread. There are three potential explanations for the lack of a clear relationship between precipitation and area burned. First, our study area has relatively low precipitation during every year of the fire season and fuel moisture is usually at critical levels during fire season so that ignition and wind events are the key determinates of burned area. It is also possible that the field capacity of soils in Xilingol grasslands is large enough that fuel moisture does not decrease because plants have sufficiently deep roots to keep fuel moisture at normal levels even during drought. The third possibility is

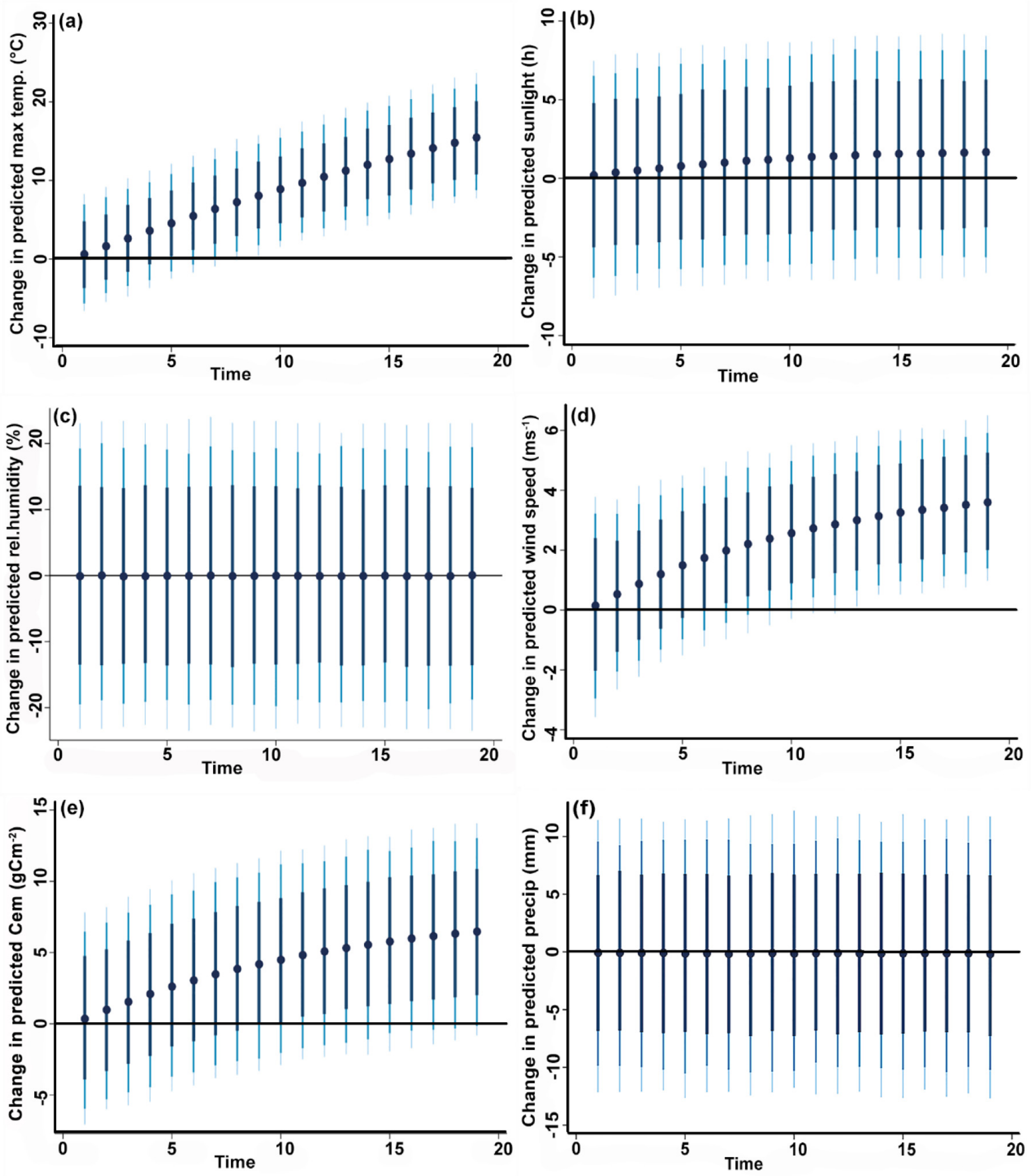

Fig 7. Plots of the dynamic stimulated ARDL model. (a) change in predicted maximum temperature on area burned (b) change in predicted sunlight on area burned (c) change in predicted humidity on area burned (d) change in predicted wind speed on area burned (e) change in predicted carbon emissions on area burned (f) change in predicted precipitation on area burned. Dots represent average predicted value while dark blue to light blue lines denote 75, 90 and 95% confidence intervals. (For interpretation of the references to color in this figure legend, the reader is referred to the web version of this article.).

https://doi.org/10.1371/journal.pone.0229894.g007

that our statistical approach is unable to accurately estimate the precipitation response because there is insufficient variation in precipitation over the 18-year time series examined.

Other studies (e.g. [14]) failed to find strong correlations between area burned and wind speed, possibly because of a shorter climate record examined (2000–2014 instead of 2001–2018) or different statistical methodology. Existing literature–[6, 14, 37] often ignore lag selection in statistical models, which may result in spurious regressions.

Table 5. Error correction representation for dynamic simulations of ARDL models.

	Observed		Predicted	
**Regressor**	**Coefficient**	**Prob.**	**Coefficient**	**Prob.**
DLn Wind speed	6.143 **	0.001	7.806**	0.001
DLn T_{max}	0.046	0.871	0.524**	0.001
DLn T_{min}	0.241	0.461	0.044	0.541
DLn Rel.humidity	0.232	0.421	0.031	0.611
DLn Precip	0.620	0.232	0.066	0.441
DLn Sunlight	2.447	0.001	2.888	0.451
DLn Cem	1.832 **	0.001	1.866**	0.001
ECM (-1)	-0.611 **	0.001	-0.626**	0.001
R^2	0.621		R^2	0.632
Adj. R^2	0.630		Adj. R^2	0.649
Number of simulations.	5000		Number of simulations.	5000
Sum squared Res.	0.006		Sum squared Res.	0.061
Durbin-Watson-stat	2.161		Durbin-Watson-stat	2.180

Notes: ARDL (1,1,1,1,1,0,1) was selected based on the Schwarz Bayesian Criterion. The dependent variable was Ln (grassland area burned) with 832 observations over the period 2001 to 2018.

** denotes 5% level of significance

https://doi.org/10.1371/journal.pone.0229894.t005

Although we demonstrate an important influence of climate on area burned across Xilingol, anthropogenic ignitions, urbanization, agriculture, and management practices may account for substantial variability in fire occurrence and pattern [38, 39]. Extreme wind events occur in northern China on a regular basis, but do not regularly result in large fire events. Major fires are dependent on the juxtaposition of these weather events with anthropogenic ignitions. Over 95% of ignitions are due to humans [4, 16], and as populations increase, we expect a greater chance of ignitions during severe fire weather conditions.

Our research suggests that the combination of increasing anthropogenic pressure on grasslands in concert with continued warming temperatures will likely increase burning in the northern China steppe, which may have significant effects to the livestock industry and conservation efforts [7, 12]. Rehabilitation and following fire in arid and semi-arid landscapes require significant time and expense. Our research demonstrates that there may be climatic thresholds past which point rising summer temperature and high wind speed events could lead to abrupt increases in area burned. The response of wind speed related to grassland area burned is the

Table 6. Monthly correlation between grassland area burned and climatic variables from 2001 to 2018.

Months	T_{max}	T_{min}	Precip	Rel.humidity	Wind	Cem	Sunlight
FMA	0.418	0.011	0.038	0.034	0.068	0.434	0.019
	(0.01)	(0.211)	(0.189)	(0.313)	(0.401)	(0.01)	(0.311)
MJJ	0.436	0.118	0.149	0.382	0.881	0.461	0.049
	(0.01)	(0.168)	(0.031)	(0.041)	(0.01)	(0.01)	(0.129)
ASO	0.488	0.014	0.021	0.031	0.841	0.421	0.632
	(0.01)	(0.506)	(0.563)	(0.865)	(0.01)	(0.01)	(0.01)

Abbreviations: February, March, April (FMA), May, June, July (MJJ), August, September, October (ASO), monthly average maximum temperature (T_{max}), monthly average minimum temperature (T_{min}), monthly precipitation (Precip), monthly average relative humidity (Humidity), monthly average wind speed (Wind), Carbon emission (Cem), monthly average sunlight (Sunlight). The top statistic in each cell is Pearson's r and the bottom parenthesis statistic denotes the probability.

https://doi.org/10.1371/journal.pone.0229894.t006

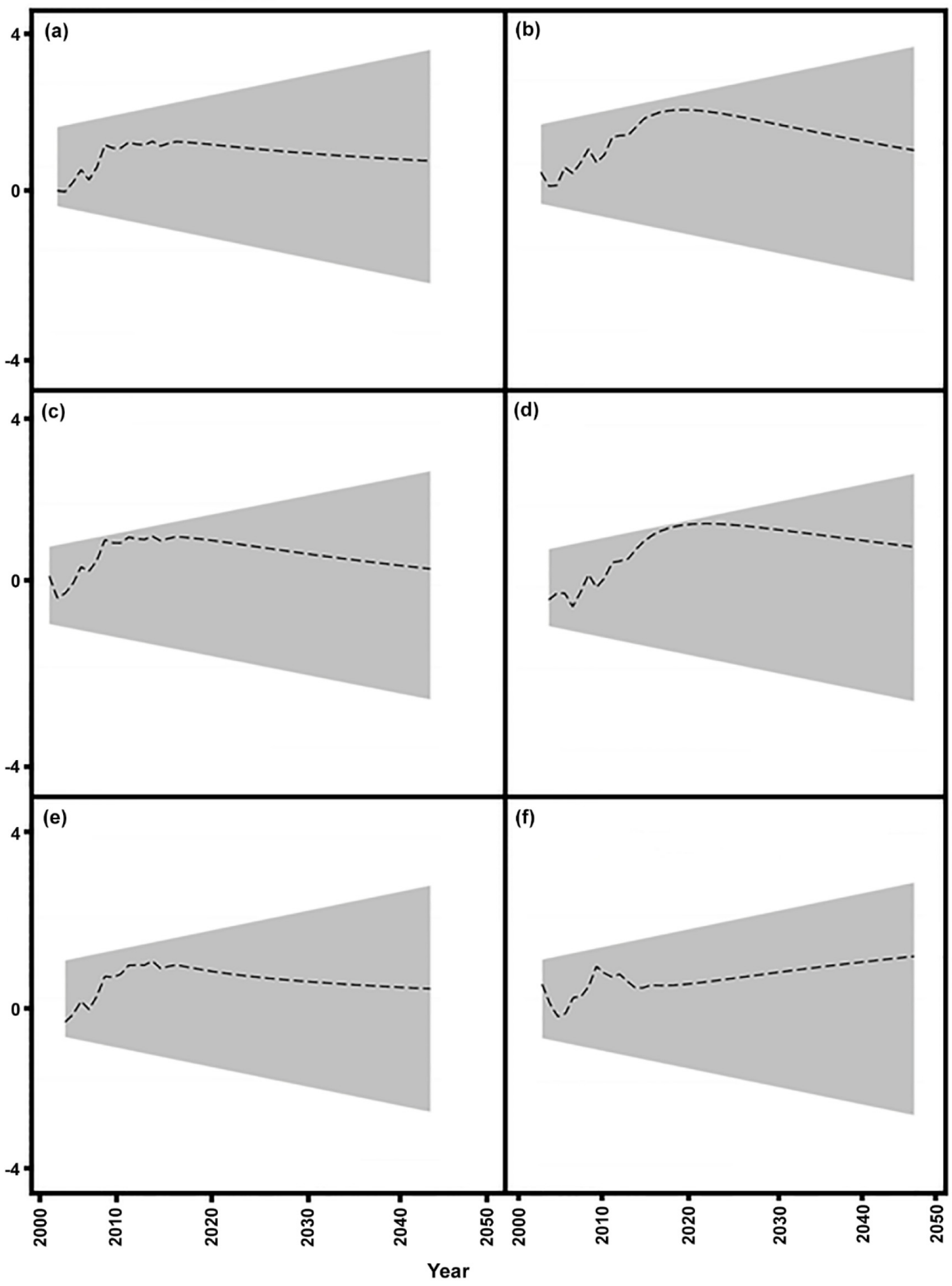

Fig 8. Plots of the cumulative sum of recursive residuals for the dynamic stimulated ARDL model. (a) maximum temperature (b) sunlight (c) humidity (d) wind speed (e) carbon emissions (f) precipitation. The recursive CUSUM plots within the 95% confidence bands confirm the stability of the estimated models.

https://doi.org/10.1371/journal.pone.0229894.g008

most critical threshold, suggesting that change in intensity of wind speed is particularly impactful.

Conclusion

Anthropogenic climate change along with an increase in the human population is likely to significantly increase the impact of fire on the globally important grassland ecosystems of Xilingol. This study successfully utilized a dynamic simulated Autoregressive Distributed Lag (ARDL) model to determine the climate variables that have the greatest effect on the area of grassland burnt. Our results indicated that many factors predicted to have an influence of the area burned—such as precipitation—were not as influential as expected. The most important factors influencing area burned are maximum temperature and wind speed. Although our results indicate that an increase in area burned is inevitable, the fire environment is not independent of human activities and changes in fire pattern will also depend on human action, government policy, and social goals.

Supporting information

S1 File. Datasets.
(RAR)

Author Contributions

Methodology: Samuel Asumadu Sarkodie.

Supervision: Jiquan Zhang.

Writing – original draft: Ali Hassan Shabbir.

Writing – review & editing: James D. Johnston, James A. Lutz, Xingpeng Liu.

References

1. Holden ZA, Swanson A, Luce CH, Jolly WM, Maneta M, Oyler JW, et al. Decreasing fire season precipitation increased recent western US forest wildfire activity. Proceedings of the National Academy of Sciences. 2018; 115(36):E8349–E57.
2. Gillett NP, Weaver AJ, Zwiers FW, et al. Detecting the effect of climate change on Canadian forest fires Geo physical Research Letters. 2004;31.
3. Collins L, Bennett AF, Leonard SW, Penman TD. Wildfire refugia in forests: Severe fire weather and drought mute the influence of topography and fuel age. Global Change Biology. 2019; 25(11):3829–43. https://doi.org/10.1111/gcb.14735 PMID: 31215102
4. Danneyrolles V, Dupuis S, Fortin G, Leroyer M, de Römer A, Terrail R, et al. Stronger influence of anthropogenic disturbance than climate change on century-scale compositional changes in northern forests. Nature communications. 2019; 10(1):1–7.
5. Syphard AD, Rustigian-Romsos H, Mann M, Conlisk E, Moritz MA, Ackerly D. The relative influence of climate and housing development on current and projected future fire patterns and structure loss across three California landscapes. Global Environmental Change. 2019; 56:41–55.
6. Abatzoglou JT, Williams AP, Boschetti L, Zubkova M, Kolden CA. Global patterns of interannual climate–fire relationships. Global change biology. 2018; 24(11):5164–75. https://doi.org/10.1111/gcb.14405 PMID: 30047195
7. Ferrara C, Marchi M, Carlucci M, Mavrakis A, Corona P, Salvati L. The 2007 crisis and Greek wildfires: a multivariate analysis of suppression times. Environmental monitoring and assessment. 2018; 190(12):714. https://doi.org/10.1007/s10661-018-7086-4 PMID: 30417241
8. Parks SA, Holsinger LM, Panunto MH, Jolly WM, Dobrowski SZ, Dillon GK. High-severity fire: evaluating its key drivers and mapping its probability across western US forests. Environmental research letters. 2018; 13(4):044037.

9. Pellegrini AF, Ahlström A, Hobbie SE, Reich PB, Nieradzik LP, Staver AC, et al. Fire frequency drives decadal changes in soil carbon and nitrogen and ecosystem productivity. Nature. 2018; 553 (7687):194–8. https://doi.org/10.1038/nature24668 PMID: 29227988
10. Keeley JE, Syphard AD. Different historical fire–climate patterns in California. International Journal of Wildland Fire. 2017; 26(4):253–68.
11. Syphard AD, Keeley JE, Pfaff AH, Ferschweiler K. Human presence diminishes the importance of climate in driving fire activity across the United States. Proceedings of the National Academy of Sciences. 2017; 114(52):13750–5.
12. Mann ML, Batllori E, Moritz MA, Waller EK, Berck P, Flint AL, et al. Incorporating anthropogenic influences into fire probability models: Effects of human activity and climate change on fire activity in California. PLoS One. 2016; 11(4):e0153589. https://doi.org/10.1371/journal.pone.0153589 PMID: 27124597
13. Parks SA, Miller C, Abatzoglou JT, Holsinger LM, Parisien M-A, Dobrowski SZ. How will climate change affect wildland fire severity in the western US? Environmental Research Letters. 2016; 11(3):035002.
14. Liu Y, Kochanski A, Baker K, Mell W, Linn R, Paugam R, et al. Fire behavior and smoke modeling: Model improvement and measurement needs for next-generation operational smoke prediction systems. Int J Wildland Fire. 2019.
15. Shabbir AH, Zhang J, Liu X, Lutz JA, Valencia C, Johnston JD. Determining the sensitivity of grassland area burned to climate variation in Xilingol, China, with an autoregressive distributed lag approach. International Journal of Wildland Fire. 2019; 28(8):628–39.
16. Davis KT, Dobrowski SZ, Higuera PE, Holden ZA, Veblen TT, Rother MT, et al. Wildfires and climate change push low-elevation forests across a critical climate threshold for tree regeneration. Proceedings of the National Academy of Sciences. 2019; 116(13):6193–8.
17. Murphy BP, Prior LD, Cochrane MA, Williamson GJ, Bowman DM. Biomass consumption by surface fires across Earth's most fire prone continent. Global change biology. 2019; 25(1):254–68. https://doi.org/10.1111/gcb.14460 PMID: 30270480
18. Andela N, Morton DC, Giglio L, Paugam R, Chen Y, Hantson S, et al. The Global Fire Atlas of individual fire size, duration, speed and direction. Earth System Science Data. 2019; 11(2).
19. Zubkova M, Boschetti L, Abatzoglou JT, Giglio L. Changes in Fire Activity in Africa from 2002 to 2016 and Their Potential Drivers. Geophysical Research Letters. 2019; 46(13):7643–53.
20. Grant T, Lebo MJ. Error correction methods with political time series. Political Analysis. 2016; 24(1):3–30.
21. Petersen MA. Estimating standard errors in finance panel data sets: Comparing approaches. The Review of Financial Studies. 2009; 22(1):435–80.
22. Williams LK, Whitten GD. But wait, there's more! Maximizing substantive inferences from TSCS models. The Journal of Politics. 2012; 74(3):685–93.
23. Bekun FV, Alola AA, Sarkodie SA. Toward a sustainable environment: Nexus between CO2 emissions, resource rent, renewable and nonrenewable energy in 16-EU countries. Science of the Total Environment. 2019; 657:1023–9. https://doi.org/10.1016/j.scitotenv.2018.12.104 PMID: 30677870
24. Engle RF. Autoregressive conditional heteroscedasticity with estimates of the variance of United Kingdom inflation. Econometrica: Journal of the Econometric Society. 1982:987–1007.
25. Mansfield ER, Helms BP. Detecting multicollinearity. The American Statistician. 1982; 36(3a):158–60.
26. Durbin J, Watson GS. Testing for serial correlation in least squares regression: I. Biometrika. 1950; 37 (3/4):409–28.
27. Andrews DW, Monahan JC. An improved heteroskedasticity and autocorrelation consistent covariance matrix estimator. Econometrica: Journal of the Econometric Society. 1992:953–66.
28. Jordan S, Philips AQ. Cointegration testing and dynamic simulations of autoregressive distributed lag models. The Stata Journal. 2018; 18(4):902–23.
29. Dickey DA, Fuller WA. Distribution of the estimators for autoregressive time series with a unit root. Journal of the American statistical association. 1979; 74(366a):427–31.
30. Gujarati DN, Porter D. Basic Econometrics Mc Graw-Hill International Edition. 2009.
31. Brown RL, Durbin J, Evans JM. Techniques for testing the constancy of regression relationships over time. Journal of the Royal Statistical Society: Series B (Methodological). 1975; 37(2):149–63.
32. Pesaran MH, Shin Y, Smith RJ. Bounds testing approaches to the analysis of level relationships. Journal of applied econometrics. 2001; 16(3):289–326.
33. Johansen S. Statistical analysis of cointegration vectors. Journal of economic dynamics and control. 1988; 12(2–3):231–54.

34. Sarkodie SA, Strezov V, Weldekidan H, Asamoah EF, Owusu PA, Doyi INY. Environmental sustainability assessment using dynamic autoregressive-distributed lag simulations—nexus between greenhouse gas emissions, biomass energy, food and economic growth. Science of the total environment. 2019; 668:318–32. https://doi.org/10.1016/j.scitotenv.2019.02.432 PMID: 30852209

35. Kripfganz S, Schneider DC, editors. ardl: Stata module to estimate autoregressive distributed lag models. Stata Conference, Chicago, July; 2016.

36. Trauernicht C. Vegetation—Rainfall interactions reveal how climate variability and climate change alter spatial patterns of wildland fire probability on Big Island, Hawaii. Science of the total environment. 2019; 650:459–69. https://doi.org/10.1016/j.scitotenv.2018.08.347 PMID: 30199690

37. Littell JS, Peterson DL, Riley KL, Liu Y, Luce CH. A review of the relationships between drought and forest fire in the United States. Global change biology. 2016; 22(7):2353–69. https://doi.org/10.1111/gcb.13275 PMID: 27090489

38. Yao J, Zhang X, Murray AT. Location optimization of urban fire stations: Access and service coverage. Computers, Environment and Urban Systems. 2019; 73:184–90.

39. Zhou Y, Bu R, Zhang X, Fan C, Gong J. Performance evaluation of water mist fire suppression: A clean and sustainable fire-fighting technique in mechanically-ventilated place. Journal of cleaner production. 2019; 209:1319–31.
